# Indacaterol and glycopyrronium versus indacaterol on body plethysmography measurements in COPD—a randomised controlled study

**DOI:** 10.1186/s12931-016-0498-1

**Published:** 2017-01-11

**Authors:** Joerg Salomon, Daiana Stolz, Guido Domenighetti, Jean-Georges Frey, Alexander J. Turk, Andrea Azzola, Thomas Sigrist, Jean-William Fitting, Ulrich Schmidt, Thomas Geiser, Corinne Wild, Konstantinos Kostikas, Andreas Clemens, Martin Brutsche

**Affiliations:** 1 Lung Centre Salem-Spital, Bern, Switzerland; 2 University Hospital Basel, Basel, Switzerland; 3 Regional Hospital La Carità, Locarno, Switzerland; 4 Hospital du Valais, Sion, Switzerland; 5 Hospital, Zürcher Rehazentrum Wald, Wald, Switzerland; 6 Regional Hospital Civico, Lugano, Switzerland; 7 Hospital, Klinik Barmelweid, Barmelweid, Switzerland; 8 Lausanne University Hospital, Lausanne, Switzerland; 9 Kliniken Valens, Rehabilitation Centre, Walenstadtberg, St. Gallen, Switzerland; 10 University Hospital of Bern, Bern, Switzerland; 11 Novartis Pharma Schweiz AG, Rotkreuz, Switzerland; 12 Novartis Pharma AG, Basel, Switzerland; 13 Cantonal Hospital, St. Gallen, Switzerland

**Keywords:** COPD, Indacaterol, Glycopyrronium, Spirometry, Body plethysmography

## Abstract

**Background:**

Dual bronchodilator therapy is recommended for symptomatic patients with chronic obstructive pulmonary disease (COPD). There are limited data on effects of a combination of two long-acting bronchodilators on lung function including body plethysmography.

**Methods:**

This multicentre, randomised, double-blind, single-dose, cross-over, placebo-controlled study evaluated efficacy and safety of the free combination of indacaterol maleate (IND) and glycopyrronium bromide (GLY) versus IND alone on spirometric and body plethysmography parameters, including inspiratory capacity (IC), forced expiratory volume in 1 s (FEV_1_), forced vital capacity (FVC), total lung capacity (TLC) and airway resistance (Raw) in moderate-to-severe COPD patients.

**Results:**

Seventy-eight patients with FEV_1_ % pred. (mean ± SD) 56 ± 13% were randomised. The combination of IND + GLY versus IND presented a numerically higher peak-IC (Δ = 0.076 L, 95% confidence interval [CI]: −0.010 – 0.161 L; *p* = 0.083), with a statistically significant difference in mean IC over 4 h (Δ = 0.054 L, 95%CI 0.022 – 0.086 L; *p* = 0.001). FEV_1_, FVC and Raw, but not TLC, were consistently significantly improved by IND + GLY compared to IND alone. Safety profiles of both treatments were comparable.

**Conclusion:**

The free combination of IND + GLY improved lung function parameters as evaluated by spirometry and body plethysmography, with a similar safety profile compared to IND alone.

**Trial registration:**

NCT01699685

**Electronic supplementary material:**

The online version of this article (doi:10.1186/s12931-016-0498-1) contains supplementary material, which is available to authorized users.

## Background

Static lung hyperinflation is one of the significant challenges in patients with COPD. It is characterised by a decrease in the elastic recoil of the lungs with a premature closure of small airways leading to air trapping. The impact on lung function parameters is expressed by an increase in functional residual capacity (FRC) and a progressive decrease in inspiratory reserve volume and inspiratory capacity (IC). During exercise, dynamic compression of the airways intensifies and this results in increased dynamic hyperinflation, leading to further exercise limitation [1]. The major clinically relevant mechanism of action of long-acting bronchodilators in COPD is related to the reduction of hyperinflation [1–5], which can be assessed by improvements in IC [6]. Whereas short-acting bronchodilators are used for immediate relief from symptoms, one or more long-acting bronchodilators (long-acting β_2_-agonists [LABAs], e.g., indacaterol maleate [IND], and long-acting muscarinic antagonists [LAMAs], e.g., glycopyrronium bromide [GLY]) are recommended for long-term maintenance therapy in patients with moderate-to-severe COPD [7]. Since LABAs and LAMAs have different mechanisms of action, they may exert additive bronchodilation effects when used together. This suggests that IND and GLY could be used in combination to optimise and maximise bronchodilation in patients with COPD whose needs are not adequately met by LABA or LAMA monotherapy [8–10]. However, there are limited data on the effects of a combination of two long-acting bronchodilators on body plethysmography lung function parameters in patients with COPD [11].

In this study we evaluated the efficacy and safety of the free combination of IND + GLY versus IND alone on lung function parameters evaluated by body plethysmography, including inspiratory capacity (IC), forced expiratory volume in 1 s (FEV_1_), forced vital capacity (FVC), total lung capacity (TLC) and airway resistance (Raw), in patients with moderate-to-severe COPD.

## Methods

### Study population

The study was conducted in 11 centres in Switzerland between November 2012 and June 2014, and included a total of 78 eligible patients who were randomised to one of two treatment sequences. The study protocol was reviewed and approved by institutional review boards and ethics committees.

Eligible patients were adults aged ≥40 years with a diagnosis of moderate or severe COPD according to GOLD criteria [12] who had signed an informed consent form, and fulfilling the following: smoking history of at least 10 pack-years [both current and ex-smokers]; post-bronchodilator FEV_1_ <80% and ≥30% of the predicted value, and post-bronchodilator FEV_1_/FVC <70%. The main exclusion criteria were COPD exacerbations requiring systemic glucocorticoid treatment or antibiotics and/or hospitalisation or a history of respiratory tract infection within 6 weeks prior to screening, concomitant pulmonary disease other than COPD, history of asthma or lung cancer, a known history of alpha-1 antitrypsin deficiency, or a history of hypersensitivity to any of the study medications or to medications from similar drug classes.

### Study design and treatment

**Fig. 1:**
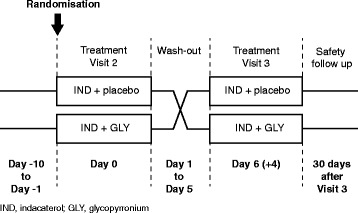
Study design

This was a multicentre, randomised, double-blind, single-dose, cross-over, placebo-controlled study to assess the effect of a single-dose combination of inhaled IND (150 μg) + GLY (50 μg) versus inhaled IND (150 μg) + placebo (corresponding GLY placebo) on static hyperinflation (Fig. 1). Patients had lung function assessments (spirometry) at each study visit and body plethysmography at Visits 2 and 3. Safety assessments included physical examinations, vital signs, and monitoring of adverse events (AEs) and serious adverse events (SAEs). All patients prematurely withdrawing from the study underwent study completion evaluations.

### Study objectives

The primary objective was to demonstrate superiority of a single dose of the combined inhalation of IND + GLY versus IND alone on peak-IC, defined as the maximum value within 4 h of inhalation. The key secondary objective was to compare the efficacy of IND + GLY versus IND in terms of FEV_1_ over 4 h (30, 60, 120, 180 and 240 min) post dosing. Other secondary objectives were to compare the efficacy of IND + GLY versus IND on IC, FVC, and airway resistance (Raw) over 4 h (30, 60, 120, 180 and 240 min) after dosing.

### Statistical analysis

#### Sample size calculation

With regard to peak-IC, a sample size of 69 patients was expected to provide 80% power to detect a difference of 0.12 L in IC at peak between the groups, assuming a standard deviation of differences of 0.35 L (test level α = 0.025 one-sided or α = 0.05 two-sided). Assuming a dropout rate of approximately 10%, a total of ~78 patients had to be randomised to ensure that at least 70 patients completed the study. Regarding FEV_1_, a sample size of 70 patients provided 99% power to detect a difference of 0.18 L in FEV_1_ mean values between the groups.

The intention to treat (ITT, full analysis set [FAS]) population consisted of all randomised patients who received at least one dose of study medication and had at least one post-baseline assessment of the primary efficacy variable. The per-protocol (PP) population consisted of all patients in the ITT population without major protocol violations or who discontinued the study due to treatment-related reasons. A supportive analysis on the PP population was performed for the primary endpoint peak-IC and the key secondary endpoint FEV_1_. The safety population (full analysis set; FAS) was defined as all randomised patients who received at least one dose of study medication with at least one post-baseline safety assessment.

Study endpoints were analysed by an analysis of covariance (ANCOVA) model with treatment sequence (AB or BA) and treatment as fixed effects, the lung function parameter as a covariate and patient as a random effect. Treatment effect was estimated as the contrast of the treatment effect in the statistical model and presented as point estimates and corresponding 95% two-sided confidence intervals (CIs). The null hypothesis for the primary analysis was that combination of IND + GLY is not superior to IND alone regarding the lung function parameters. The alternative hypothesis was that treatment with a combination of IND + GLY is superior to IND alone. The null hypothesis was rejected in favour of the alternative hypothesis if the 95% CI of the least squares means treatment contrast of the difference “combination therapy — single therapy” was greater than 0 in its entirety. This corresponds to a planned alpha error of 5% two-sided or 2.5% one-sided. An interim analysis was performed after 20 patients had completed Visit 3. No adjustments were needed.

## Results

**Fig. 2:**
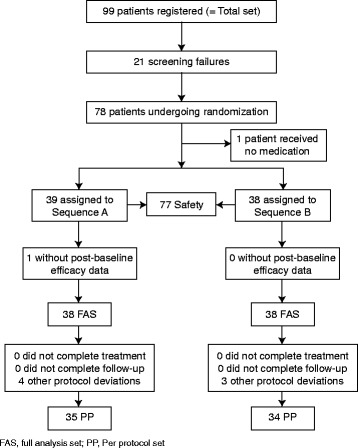
Disposition of patients

The mean ± SD age of the patients was 64.8 ± 8.4 years (Table 1), 59.2% were male, all Caucasian, and 24 (31.2%) current smokers. Mean time since COPD diagnosis was 5.2 ± 5.2 years. The mean FEV_1_% predicted was 56 ± 13 and 38.7% of patients had a GOLD stage of III or above. The mean total lung capacity (TLC) was 120.68 ± 18.75% pred. and the mean Raw was 210.99 ± 117.11% pred. The patient disposition and randomisation is given in Fig. 2.

**Table 1:** Demography and baseline characteristics (ITT population, *N* = 76)

		Mean (SD), *N* = 76
Age at informed consent, years		64.80 (8.39)
Height, cm		168.22 (8.55)
Weight, kg		75.45 (15.52)
BMI, kg/m^2^		26.65 (5.05)
Smoking history Number of pack-years, years		50.13 (23.28)
Years since COPD diagnosis		5.17 (5.24)
Age at COPD diagnosis, years		60.16 (10.96)
FEV_1_ % predicted		56.09 (13.28)
FEV_1_*, L		1.50 (0.45)
FVC*, L		2.95 (0.89)
IC, L		2.45 (0.69)
Total Lung Capacity (TLC), L		7.13 (1.42)
TLC % of predicted normal value		120.68 (18.75)
Airway Resistance (Raw)		6.73 (3.19)
Raw % of predicted normal value		210.99 (117.11)
Hyperinflation IC/TLC		0.35 (0.09)
		N (%)
Gender	Male	45 (59.2)
	Female	31 (40.8)
Number of patients with current medical condition	CAD	7 (9.2)
	Hypertension	29 (38.2)
	Diabetes mellitus	4 (5.3)
Number of patients according COPD GOLD-stage	Stage I	8 (10.5)
	Stage II	31 (40.8)
	Stage III	30 (39.5)
	Stage IV	7 (9.2)

*ITT* intention to treat, *N/n* number of patients, *BMI* body mass index, *SD* standard deviation, *COPD* chronic obstructive pulmonary disease, *FEV*_*1*_ forced expiratory volume in 1 s, *FVC* forced vital capacity, *IC* inspiratory capacity, *CAD* coronary artery disease, GOLD stage defined as: stage I = FEV_1_/FVC <70% and FEV_1_ ≥80% predicted; stage II = FEV_1_/FVC <70% and 50% ≤FEV_1_ <80% predicted; stage III = FEV_1_/FVC <70% and 30% ≤FEV1 <50% predicted; stage IV = FEV_1_/FVC <70% and FEV_1_ <30% predicted

^*^ *N* = 75

**Fig. 3:**
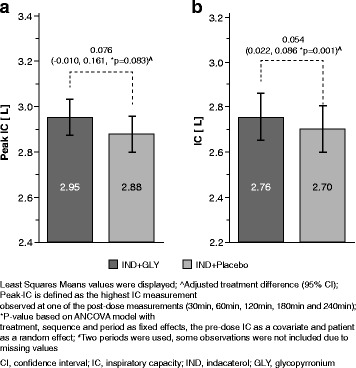
Improvements in **a** Peak Inspiratory Capacity (peak-IC) [L] (*N* = 74) and (**b**) Mean inspiratory Capacity [L] (*N* = 77) by IND + GLY versus IND alone

**Fig. 4:**
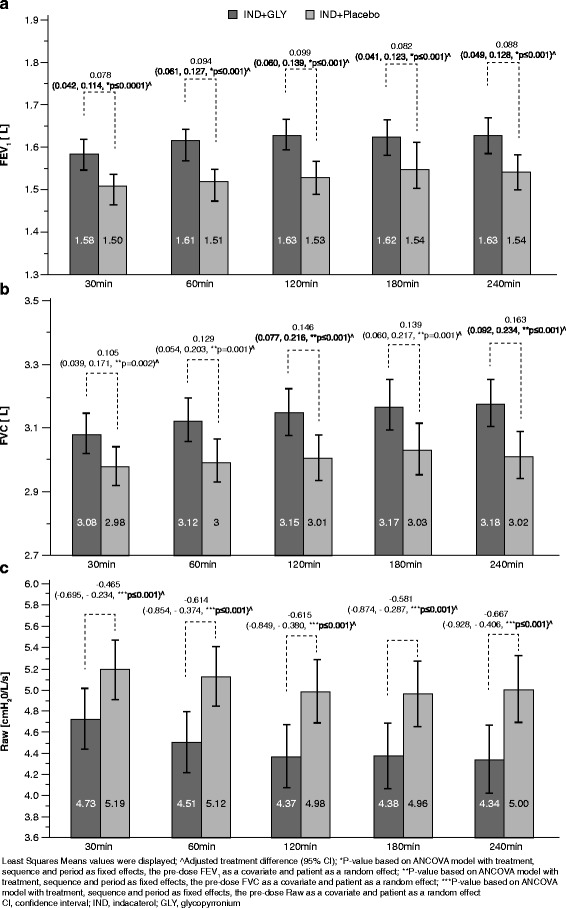
**a** Forced expiratory volume in 1 s (FEV1) [L] over time (ITT population, *N* = 77); **b** Forced vital capacity (FVC) [L] (*N* = 77); **c** Airway resistance (Raw) [cmH2O/L/s] (*N* = 77)

The combination of IND + GLY versus IND presented a numerically higher peak-IC (2.95 L versus 2.88 L), with an adjusted treatment difference (Δ) of 0.076 L (95% −0.010 – 0.161 L; *p* = 0.083) (Fig. 3a). IND + GLY presented also a statistically significant difference in mean IC over 4 h versus IND (2.76 L versus 2.70 L; Δ = 0.054 L, 95% CI 0.022 – 0.086 L; *p* = 0.001) (Fig. 3b). FEV_1_, FVC and Raw, but not TLC, were significantly improved by IND + GLY compared to IND alone. A statistically significant adjusted treatment difference in FEV_1_ was noted at all time points in favour of IND + GLY treatment (*p* <0.001 for all comparisons), reaching a peak difference of Δ = 0.099 L (95%CI 0.060 – 0.139 L) at 120 min post-dose (Fig. 4a). Similarly, IND + GLY resulted in higher FVC mean values at all time points after a single-dose inhalation (*p* <0.01 for all comparisons), reaching a peak difference of Δ = 0.163 L (95%CI 0.092 – 0.234 L) at 240 min post-dose (Fig. 4b). Raw measurements were consistently lowered by IND + GLY treatment at all time points after the single-dose inhalation (p <0.001 for all comparisons), reaching a peak difference of Δ = -0.667 cmH_2_O/L/sec (95%CI -0.928 – -0.406 cmH2O/L/sec) at 240 min post-dose (Fig. 4c), in favour of dual bronchodilation (*p* ≤0.001). There were no differences in TLC between the study treatments.

### Safety

Eight (10.4%) patients experienced treatment-emergent adverse events (TEAEs) (Table 2). No patient died in the course of the study or experienced any treatment-emergent SAE. According to the investigators’ assessment, a relation to study medication was not suspected for any of the TEAEs. The intensity of TEAEs was mostly mild (6 patients) or moderate (2 patients). Prior to the first dose of study medication, one patient experienced atrial fibrillation of moderate intensity. In conclusion, the treatments were well tolerated with a good safety profile.

**Table 2:** Incidence of TEAEs by primary system organ class (safety population, *N* = 77)

	Combined treatment (IND + GLY)	IND alone (IND + placebo)	All
Primary system organ class	*N* = 77, n (%)	*N* = 77, n (%)	*N* = 77, n (%)
Number (%) of patients with at least one AE	5 (6.5)	3 (3.9)	8 (10.4)
Investigations	1 (1.3)	2 (2.6)	3 (3.9)
Respiratory, thoracic & mediastinal disorders	2 (1.3)	1 (1.3)	3 (3.9)
Infections & infestations	1 (1.3)	0	1 (1.3)
Musculoskeletal and connective tissue disorders	1 (1.3)	0	1 (1.3)

*TEAEs* treatment-emergent adverse events, *IND* indacaterol, *IND + GLY* indacaterol and glycopyrronium, *N or n* number of patients, *AE* adverse event

## Discussion

In this prospective, randomised study we showed that the combination of two long-acting bronchodilators provided a greater improvement in lung hyperinflation and lung function parameters compared to a single long-acting agent. Specifically, IND + GLY provided a numerical improvement in peak-IC combined with a statistically significant difference in mean IC over 4 h compared to IND monotherapy. Additionally, the treatment with IND + GLY resulted in consistent statistically significant improvements in FEV_1_, FVC and Raw compared to IND alone. The two treatments presented a similar safety profile.

As a unique feature of the trial, the use of body plethysmography allowed us to observe the significant difference in Raw in favour for IND + GLY in this study. Raw is not frequently reported in studies evaluating the effect of bronchodilators in COPD. However, this parameter is suggested to be sensitive and to reflect airflow obstruction, particularly of the peripheral airways, more accurately than the FEV_1_/FVC ratio. In assessing the acute functional effect of bronchodilators, specific Raw change-based criteria may be preferable to FEV_1_- or FVC-based criteria, being more closely related to bronchodilator-induced improvements in lung mechanics and dyspnoea at rest [13]. Raw measurements were strongly improved by IND + GLY treatment compared to IND monotherapy at all time points after single-dose inhalation.

A possible explanation of the non-statistically significant result in SYNERGY on peak-IC might be attributed to the high variability of this measurement. This is supported by the fact that in contrast to the peak-IC measurement, the adjusted mean IC in the SYNERGY study (which included several values) presented a statistically significant difference between the two treatments. Additionally, the results of the present study are consistent with those of other published studies that have investigated the efficacy and safety of LABA/LAMA combination therapy in patients with COPD [7, 11, 14–18]. In order to allow for higher power and better generalisability of the results, we additionally evaluated with a similar analysis as in SYNERGY the peak-IC and FEV_1_ in a pooled analysis of patient-level data (*n* = 1,548) from 3 studies that evaluated the combination of IND + GLY versus IND, i.e. SYNERGY (present study), SHINE [14] and GLOW6 [7] (see details in the Additional file 1 Online Supplement). Mean adjusted peak-IC in this pooled analysis was statistically significantly higher for patients treated with IND + GLY versus IND alone (Δ = 0.075 L; 95% CI 0.040 – 0.109 L; *p* ≤0.001) (Additional file 2 Figure S1). Additionally, FEV_1_ was statistically significantly higher for IND + GLY versus IND at 30, 120 and 240 min after a single dose inhalation, with a maximal difference at 120 min (Δ = 0.094 L; 95% CI 0.076 – 0.112 L; *p* ≤0.001) (Additional file 3 Figure S2). These results further support the reduction of static hyperinflation, as expressed by IC, by a combination of two bronchodilators compared to a single agent.

The physiological and clinical significance of these results can be attributed to prolonged maximal bronchodilation that minimises air trapping and leads to effective reduction of static and dynamic lung hyperinflation. Improved IC is associated with improved exercise endurance and dyspnoea [2, 3] and potentially improved long-term outcomes. Casanova et al. showed that lung hyperinflation, as expressed by the IC/TLC ratio, is an independent predictor of mortality [19]. Furthermore, Tantucci et al. identified IC as a powerful functional predictor of all-cause and respiratory mortality and of exacerbation-related hospital admissions in patients with COPD [20].

The improvement in bronchodilation and measures of hyperinflation observed in the present study is supported by data from the BRIGHT study (IND/GLY fixed-dose combination versus placebo and tiotropium), which showed significantly improved dynamic IC, trough FEV_1_, residual volume (RV) and FRC in patients with moderate-to-severe COPD receiving IND/GLY that were accompanied by increased exercise endurance [11]. Mahler et al. showed that IND + tiotropium provided greater bronchodilation and lung deflation compared with tiotropium monotherapy [17]. To what extent these effects have a clinically significant impact on outcomes other than lung function and exercise endurance requires further evaluation. However, there is significant evidence that exacerbations, the relevant trigger for progression, are more effectively prevented by IND + GLY than by a single long-acting bronchodilator [21].

We acknowledge that there were limitations in the study. These include the cross-over study design, the short study duration, and the potentially limited patient population due to the clinical trial settings. Additionally, we need to acknowledge that in patients with severe airflow limitation, the plethysmographic Raw may be of limited validity. Finally, post hoc it became obvious that possibly the initially taken assumptions for the power calculations were overestimated, resulting in a relatively small sample size to reach statistical significance. This is supported by the results of the pooled analysis showing the statistical significance for peak-IC.

In our study all treatments were equally well tolerated and showed a good safety profile, which is also documented in multiple clinical trials and the use in clinical practice [7, 8, 10, 11, 14, 16, 17, 21].

## Conclusions

In summary, the results of the present study show that treatment with IND + GLY had a stronger beneficial effect on lung hyperinflation and airflow obstruction parameters in patients with COPD than treatment with IND alone. The treatment was well tolerated and had a good safety profile. These data support the use of dual bronchodilator therapy to not only improve airway calibre (FEV_1_) but also decrease hyperinflation and its associated negative consequences in patients with COPD.

## Additional files

Additional file 1: Online supplement. (DOCX 182 kb)
Additional file 2: Figure S1. Peak Inspiratory Capacity [L] – pooled analysis of SYNERGY, SHINE and GLOW6 (*N* = 1538)^#^. (PDF 376 kb)
Additional file 3: Figure S2. Forced expiratory volume in 1 s (FEV1) [L] – pooled analysis of SYNERGY, SHINE and GLOW6 (*N* = 1503). (PDF 371 kb)

## Abbreviations

AEs: Adverse events
ANCOVA: Analysis of covariance
CI: Confidence interval
COPD: Chronic obstructive pulmonary disease
FAS: Full analysis set
FEV_1_: Forced expiratory volume in 1 s
FRC: Functional residual capacity
FVC: Forced vital capacity
GLY: Glycopyrronium bromide
IC: Including inspiratory capacity
IND: Indacaterol maleate
IND: Indacaterol maleate
ITT: Intention to treat
LABAs: Long-acting β2-agonists
LAMAs: Long-acting muscarinic antagonists
PP: Per-protocol
SAEs: Serious adverse events
TEAEs: Treatment-emergent adverse events
TLC: Total lung capacity
