# Supplementary material for: Indacaterol and glycopyrronium versus indacaterol on body plethysmography measurements in COPD—a randomised controlled study
Source: Respir Res. 2017 Jan 11;18:13. doi: 10.1186/s12931-016-0498-1 (PMC5225517; doi:10.1186/s12931-016-0498-1)
Supplement: Additional file 1: — Online supplement. (DOCX 182 kb) [file 12931_2016_498_MOESM1_ESM.docx]

**ONLINE SUPPLEMENT**

**Indacaterol and Glycopyrronium versus Indacaterol alone on Body Plethysmography Measurements in COPD - A randomised controlled study**

Joerg Salomon^1^, Daiana Stolz^2^, Guido Domenighetti^3^, Jean-Georges Frey^4^, Alexander J. Turk^5^, Andrea Azzola^6^, Thomas Sigrist^7^, Jean-William Fitting^8^,Ulrich Schmidt^9^,Thomas Geiser^10^, Corinne Wild^11^, Konstantinos Kostikas^12^, Andreas Clemens^#12^, Martin Brutsche^13^

1 Lung Center Salem-Spital, Bern, Switzerland

2 University Hospital Basel, Basel, Switzerland

3 Regional Hospital La Carità, Locarno, Switzerland

4 Hospital du Valais, Sion, Switzerland

5 Hospital, Zürcher Rehazentrum Wald, Wald, Switzerland

6 Regional Hospital Civico, Lugano, Switzerland

7 Hospital, Klinik Barmelweid, Barmelweid, Switzerland

8 Lausanne University Hospital, Lausanne, Switzerland

9 Kliniken Valens, Rehabilitation Centre, Walenstadtberg

10 University Hospital of Bern, Bern, Switzerland

11 Novartis Pharma Schweiz AG, Rotkreuz, Switzerland

12 Novartis Pharma AG, Basel, Switzerland

13 Cantonal Hospital, St. Gallen, Switzerland

^#^Corresponding author:

Dr Andreas Clemens

Novartis Pharma AG,

WSJ.210.14.30.3A,

CH-4056 Basel, Switzerland

Phone: +41 61 6965836 / Fax: +41 61 3248001

EMAIL: andreas.clemens@novartis.com

Summary:

Indacaterol and glycopyrronium showed a stronger beneficial effect in patients with COPD than indacaterol alone.

**POOLED ANALYSIS OF 3 TRIALS WITH SIMILAR DESIGN (SYNERGY, SHINE AND GLOW6)**

**METHODS**

**Study population**

As in the SYNERGY study, all patients included in the pooled analysis were ≥40 years of age, with moderate-to-severe stable COPD (GOLD stage II or III according to the GOLD 2010 guidelines)[1], and a smoking history of ≥10 pack-years. At screening they were required to have a post-bronchodilator FEV_1_ of <80% and ≥30% of the predicted normal value. We had access to patient-level data of all trials.

**Study designs and treatment**

The designs of the studies included in the pooled analysis are described elsewhere [2, 3]. All were multicentre, randomised, double-blind trials which compared IND+GLY with IND in one of their treatment groups. Patient-level data were taken from the qualifying treatment groups and pooled. As we were concentrating in the pooled analysis on the effect of treatment-naïve patients (post-wash out, first visit measures only), the duration of each trial (SHINE, 26 weeks; GLOW6, 12 weeks) was not relevant.

**Statistical analysis**

The statistical analysis in the pooled analysis followed the same path as described for the SYNERGY trial. Missing data were reasons for exclusion of patients from the analysis for each respective endpoint.

**RESULTS**

Patient demographics in the pooled population are presented in **Table S1**. Mean adjusted peak-IC in this pooled analysis was statistically significantly higher for patients treated with IND+GLY versus IND alone (2.54 versus 2.47 L; Δ=0.075 L; 95% CI 0.040 to 0.109; p≤0.001) (**Figure S1**). Additionally, FEV_1_ was statistically significantly higher for IND+GLY versus IND at 30, 120 and 240 min after a single dose inhalation, with a maximal difference at 120 minutes (Δ=0.094 L; 95% CI 0.076 to 0.112 L; p≤0.001) (**Figure S2**).

References

1. GOLD. Global Initiative for Chronic Obstructive Lung Disease (GOLD). Global Strategy for the Diagnosis, Management and Prevention of COPD, Global Initiative for Chronic Obstructive Lung Disease GOLD 2010. Available from [www.goldcopd.com](http://www.goldcopd.com). Accessed on June, 23, 2016, 2010.

2. Bateman ED, Ferguson GT, Barnes N, Gallagher N, et al. Dual bronchodilation with QVA149 versus single bronchodilator therapy: the SHINE study. Eur Respir J 2013; 42: 1484-1494.

3. Vincken W, Aumann J, Chen H, Henley M, McBryan D, Goyal P. Efficacy and safety of coadministration of once-daily indacaterol and glycopyrronium versus indacaterol alone in COPD patients: the GLOW6 study. Int J Chron Obstruct Pulmon Dis 2014; 9: 215-228.

**TABLES**

**Table S1. Baseline characteristics (pooled analysis SYNERGY, SHINE and GLOW6; ITT population, N =1,548)**

|  | | IND  (N=772) | IND+GLY (N=776) | P value |
| --- | --- | --- | --- | --- |
|  | | Mean (SD) | Mean (SD) |  |
| Age, years | | 63.91 (8.54) | 63.86 (8.62) | 0.911 |
| Height, cm | | 168.48 (8.78) | 167.85 (8.68) | 0.151 |
| Weight, kg | | 75.06 (17.31) | 74.57 (17.25) | 0.582 |
| BMI, kg/m^2^ | | 26.34 (5.32) | 26.35 (5.25) | 0.985 |
| Smoking history Number of pack-years, year | | 45.18 (24.24) | 44.79 (23.87) | 0.750 |
| Years since COPD diagnosis | | 6.37 (5.52) | 6.32 (5.55) | 0.850 |
| Age at COPD diagnosis, years | | 57.51 (9.29) | 57.60 (9.45) | 0.851 |
| FEV_1_ % predicted | | 47.01 (13.02) | 47.28 (13.20) | 0.679 |
| FEV_1_*, L | | 1.31 (0.44) | 1.30 (0.46) | 0.862 |
| FVC*, L | | 2.84 (0.82) | 2.77 (0.81) | 0.088 |
| IC, L | | 2.18 (0.66) | 2.12 (0.67) | 0.118 |
|  | | n (%) | n (%) | p value |
| Gender | Male | 588 (76.2) | 584 (75.3) | 0.667 |
|  | Female | 184 (23.8) | 192 (24.7) |  |
| Number of patients with current medical condition | CAD | 77 (10.0) | 82 (10.6) | 0.701 |
|  | Hypertension | 363 (47.0) | 350 (45.1) | 0.449 |
|  | Diabetes mellitus | 79 (10.2) | 81 (10.4) | 0.895 |

IND: indacaterol; IND+GLY: indacaterol and glycopyrronium; N/n: number of patients; BMI: body mass index; SD: standard deviation; COPD: chronic obstructive pulmonary disease; FEV_1_: forced expiratory volume in 1 second; FVC: forced vital capacity; IC: inspiratory capacity; CAD: coronary artery disease.

**Figure S1: Peak Inspiratory Capacity [L] – pooled analysis of SYNERGY, SHINE and GLOW6 (N=1538)^#^**

**
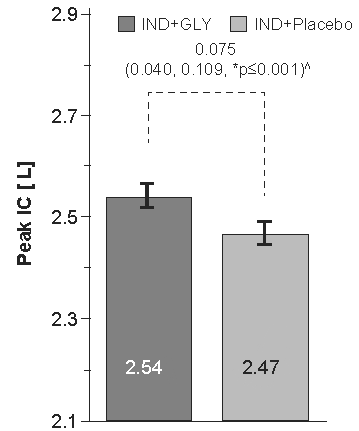
**

Least Squares Means values were displayed; ^Adjusted treatment difference (95% CI); Peak-IC is defined as the highest IC measurement observed at one of the post-dose measurements (30min, 120min and 240min); *P-value based on ANCOVA model with treatment as a fixed effect, the pre-dose IC as a covariate and patient as a random effect. ^#^Two periods were used, some observations were not included due to missing values

F**igure S2: Forced expiratory volume in 1 sec (FEV_1_) [L] – pooled analysis of SYNERGY, SHINE and GLOW6 (N=1503)**


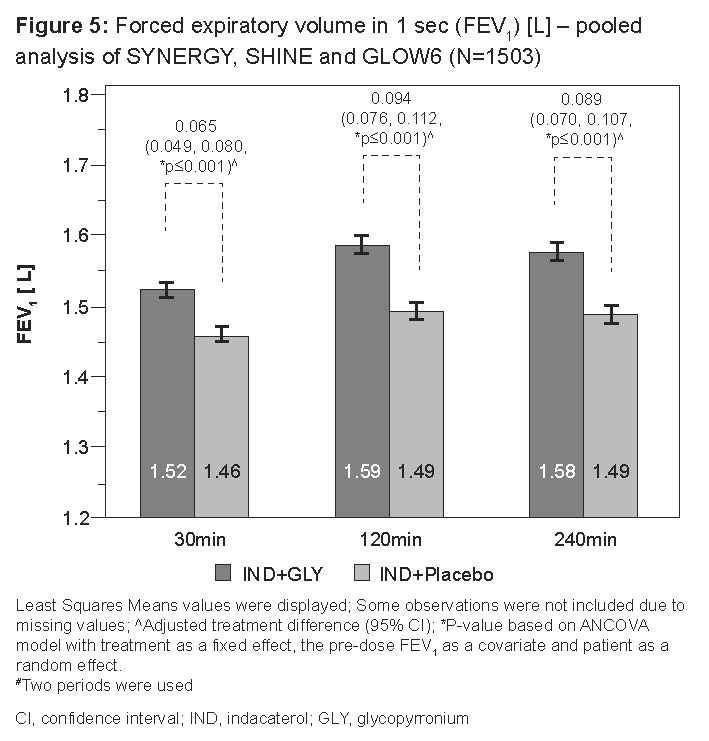


Least Squares Means values were displayed; ^Adjusted treatment difference (95% CI); *P-value based on ANCOVA model with treatment as a fixed effect, the pre-dose FEV1 as a covariate and patient as a random effect; ^#^Two periods were used, some observations were not included due to missing values

CI, confidence interval; IND, indacaterol; GLY, glycopyrronium
