# Supplementary material for: Indacaterol and glycopyrronium versus indacaterol on body plethysmography measurements in COPD—a randomised controlled study
Source: Respir Res. 2017 Jan 11;18:13. doi: 10.1186/s12931-016-0498-1 (PMC5225517; doi:10.1186/s12931-016-0498-1)
Supplement: Additional file 2: Figure S1. — Peak Inspiratory Capacity [L] – pooled analysis of SYNERGY, SHINE and GLOW6 (N = 1538)#. (PDF 376 kb) [file 12931_2016_498_MOESM2_ESM.pdf]

**Figure S1:** Peak Inspiratory Capacity [L] – pooled analysis of SYNERGY, SHINE and GLOW6 (N=1538)[#]

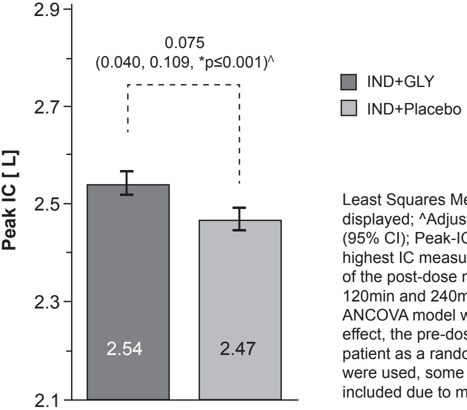


Least Squares Means values were displayed; ^Adjusted treatment difference (95% CI); Peak-IC is defined as the highest IC measurement observed at one of the post-dose measurements (30min, 120min and 240min); *P-value based on ANCOVA model with treatment as a fixed effect, the pre-dose IC as a covariate and patient as a random effect. [#]Two periods were used, some observations were not included due to missing values
