# Supplementary material for: Indacaterol and glycopyrronium versus indacaterol on body plethysmography measurements in COPD—a randomised controlled study
Source: Respir Res. 2017 Jan 11;18:13. doi: 10.1186/s12931-016-0498-1 (PMC5225517; doi:10.1186/s12931-016-0498-1)
Supplement: Additional file 3: Figure S2. — Forced expiratory volume in 1 s (FEV1) [L] – pooled analysis of SYNERGY, SHINE and GLOW6 (N = 1503). (PDF 371 kb) [file 12931_2016_498_MOESM3_ESM.pdf]

Figure S2: Forced expiratory volume in 1 sec (FEV_1) [L] – pooled analysis of SYNERGY, SHINE and GLOW6 (N=1503)

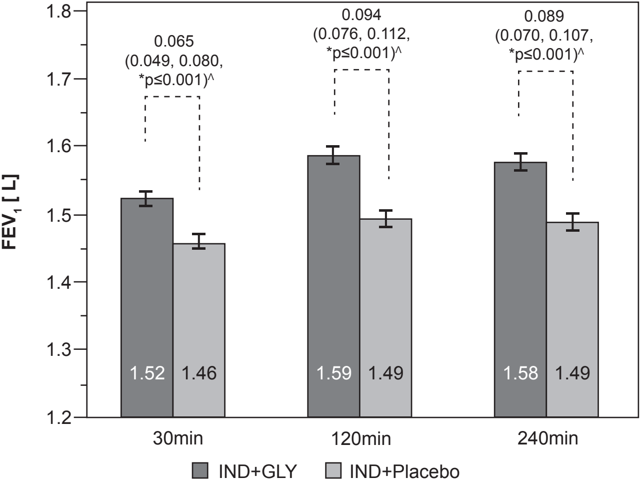

Least Squares Means values were displayed; Some observations were not included due to missing values; ^Adjusted treatment difference (95% CI); *P-value based on ANCOVA model with treatment as a fixed effect, the pre-dose FEV_1 as a covariate and patient as a random effect.

#Two periods were used

CI, confidence interval; IND, indacaterol; GLY, glycopyrronium
